# Supplementary material for: A dynamic attractor network model of memory formation, reinforcement and forgetting
Source: PLoS Comput Biol. 2023 Dec 20;19(12):e1011727. doi: 10.1371/journal.pcbi.1011727 (PMC10766193; doi:10.1371/journal.pcbi.1011727)
Supplement: S1 Text — (PDF) [file pcbi.1011727.s001.pdf]

# Supporting Information for

# A dynamic attractor network model of memory formation, reinforcement and forgetting

Marta Boscaglia[1,2], Chiara Gastaldi[3], Wulfram Gerstner[3], and Rodrigo Quian Quiroga[1,4,5,6]

1 Centre for Systems Neuroscience, University of Leicester, United Kingdom.

2 School of Psychology and Vision Sciences, University of Leicester, United Kingdom.

3 School of Computer and Communication Sciences and School of Life Sciences, École Polytechnique Fédérale de Lausanne (EPFL), Lausanne, Switzerland.

4 Hospital del Mar Medical Research Institute (IMIM), Barcelona, Spain.

5 Institució Catalana de Recerca i Estudis Avançats (ICREA), Barcelona, Spain.

6 Ruijin hospital, Shanghai Jiao Tong University School of Medicine, Shanghai, China

## Supporting Information Text

### Network activity until convergence

In order to study further the convergence of the network, we ran longer simulations (10 times longer than the ones shown in the main text) with a network of $N = 100$ units.

First, we considered the case of “Assembly evolution”, with one assembly of 10 out of $N = 100$ neurons stimulated for 70000 times with $f = 1/(60\ a.u.)$. We observe that the assembly size increases until reaching a plateau level at the size of 69 neurons (S1 Fig). Most of the evolution of the assembly (specifically, until almost 70% of the final size) takes place in the first 10% of the stimulation phase (see inset in S1 Fig, top left), which corresponds to the stimulation duration analyzed in Fig 5 of the main text. Therefore, with $f = 1/(60\ a.u.)$, the size of the stimulated assembly stabilizes without further recruiting the remaining neurons of the network. However, an assembly can also recruit all the units in the network if the frequency of stimulation is high enough. This is the case of a single assembly of 10 out of N=100 neurons stimulated with $f = 1/(30\ a.u.)$ for 100000 times (S2 Fig). Importantly, we observe that, at the time of the 100000$^{th}$ stimulation, : i) the network activity is still controlled by the external stimulation (S2 Fig, left); ii) the network connectivity (S2 Fig, right) holds meaningful information reflecting the stimulation history (i.e. one and only one assembly stimulated at relatively high frequency for relatively long time).

Since we showed that a single assembly of 10 out of N=100 neurons stimulated for 70000 times with $f = 1/(60\ a.u.)$ reaches a size of 69 neurons (S1 Fig), it is reasonable to expect that two assemblies stimulated with $f = 1/(60\ a.u.)$ would take all the neurons

of the network. We tested this, particularly to check if the condition of orthogonality between assemblies subject to uncorrelated stimuli would hold even in the extreme condition of having all the network being part of one or the other assembly. Our results show that two assemblies stimulated at $f = 1/(60\ a.u.)$ overall take the whole network. However, the two assemblies: i) reach similar sizes (S3 Fig, bottom), in line with the fact of being stimulated at the same frequency; ii) remain well separated (S3 Fig, top right), in line with the fact of being subject to uncorrelated stimuli.

Considering all this, these results further support that, in our model, the runaway dynamics of Hebbian learning [1] is successfully limited, allowing the network to deviate its activity from baseline, encode meaningful information through the change of assembly sizes and then go back to baseline after stimulation.

## Scalability of the model

In none of the simulations analyzed in Fig 11 of the main text there was overlap of neurons between different assemblies (S4 Fig).

## Stability mechanisms

The mechanisms adopted to stabilize the system (hard bounds of the synaptic weights; weight decay; synaptic normalization; divisive normalization) were all necessary to ensure the functional behavior of the system (S5 Fig).

## Comparison of assembly evolution in different experimental paradigms

In order to compare the evolution of assemblies stimulated with the same frequency but in different paradigms (namely: single-memory network; 2 patterns stimulated with the same frequency; 2 patterns stimulated with different frequencies), we compared the results shown in Figs 6A, 7A and 8A of the main text. We can observe that the sizes of assemblies stimulated with the same frequency evolved overall similarly in the different paradigms (S6A Fig). It can be noted, however, that, in case of 2 assemblies stimulated with $f = 1/(60\ a.u.)$, the assembly growth of each of the assemblies stimulated with $f = 1/(60\ a.u.)$ was slower compared to the case of 1 assembly stimulated with $f = 1/(60\ a.u.)$ and to the case of 2 assemblies stimulated with $f_1 = 1/(60\ a.u.)$ and $f_2 = 1/(120\ a.u.)$ (S6B Fig, top; ANOVA and post-hoc t-tests at three different stages: t = 1000 a.u.; t = 200000 a.u. and t = 420000 a.u.). This result seems reasonable considering that, in the case of 2 patterns stimulated with $f = 1/(60\ a.u.)$, there were two patterns stimulated with a relatively high frequency, both recruiting neurons, instead of only 1 pattern or 1 pattern with relatively high frequency and one with relatively low frequency. Regarding the evolution of the assemblies stimulated with $f = 1/(120\ a.u.)$, we found a significant difference, at the end of the simulations, in the sizes of the assemblies stimulated with $f = 1/(120\ a.u.)$ in a single-memory network compared to the case of networks with 2 patterns (S6B Fig, bottom; t-test). This result is in line with what found for the assemblies stimulated with $f = 1/(60\ a.u.)$.

**SI References**

1. Zenke F., and Gerstner W. Hebbian plasticity requires compensatory processes on multiple timescales. Phil. Trans. R. Soc. B., 372(1715):20160259, 2017. Available at **https://doi.org/10.1098/rstb.2016.0259**.
