# Supplementary figures and images for: A dynamic attractor network model of memory formation, reinforcement and forgetting
Source: PLoS Comput Biol. 2023 Dec 20;19(12):e1011727. doi: 10.1371/journal.pcbi.1011727 (PMC10766193; doi:10.1371/journal.pcbi.1011727)

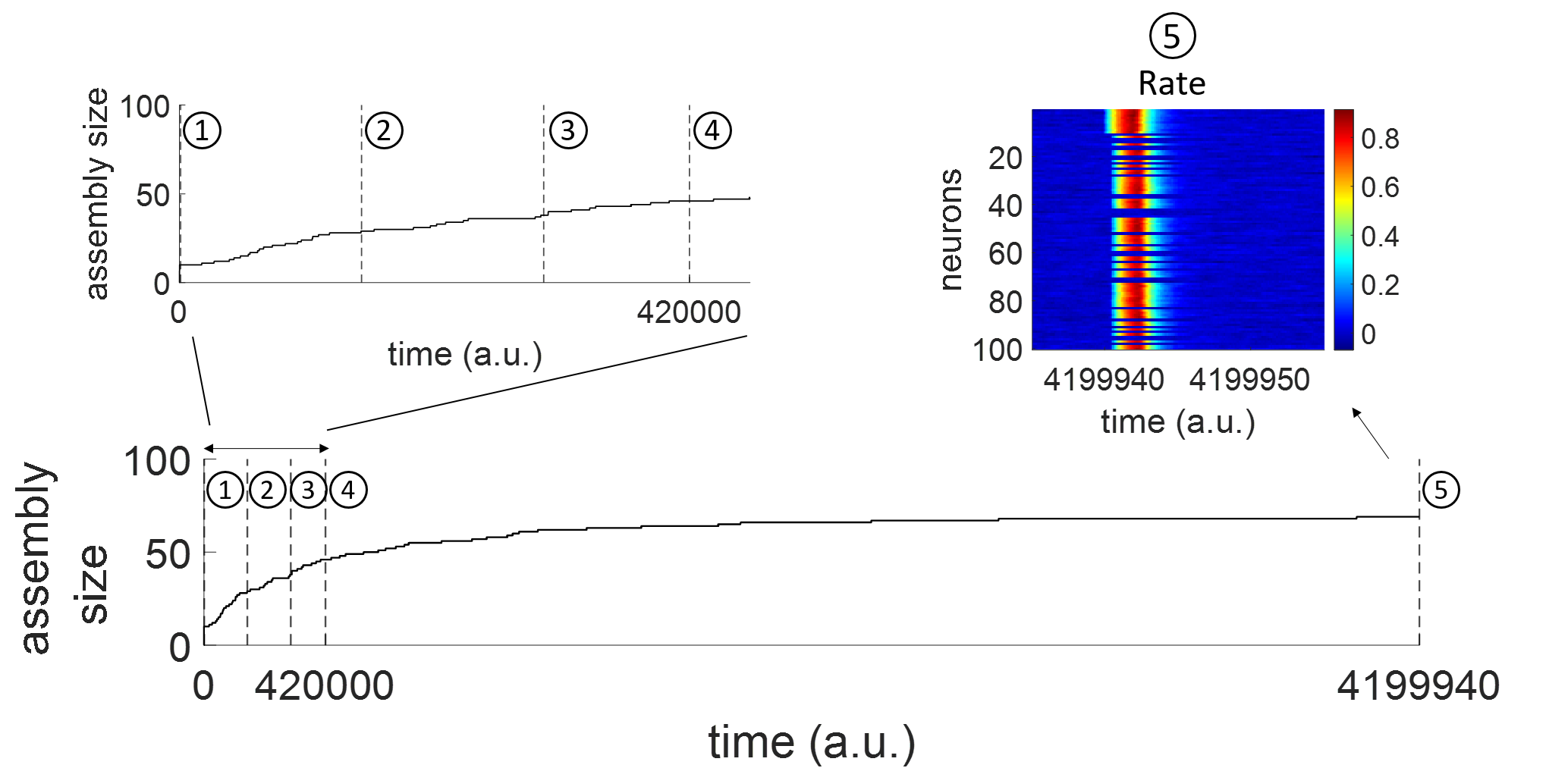

Supplement: S1 Fig — Starting at time 0 a.u., 10 neurons were stimulated for 70000 times with f = 1/(60 a.u.). Bottom: Number of assembly neurons over time. Inset, top left: zoom with enlarged time scale. Top, right: firing rate for all neurons at the time of the 70000th stimulation (the 10 directly stimulated neurons are on top). (TIF) [file pcbi.1011727.s002.tif]

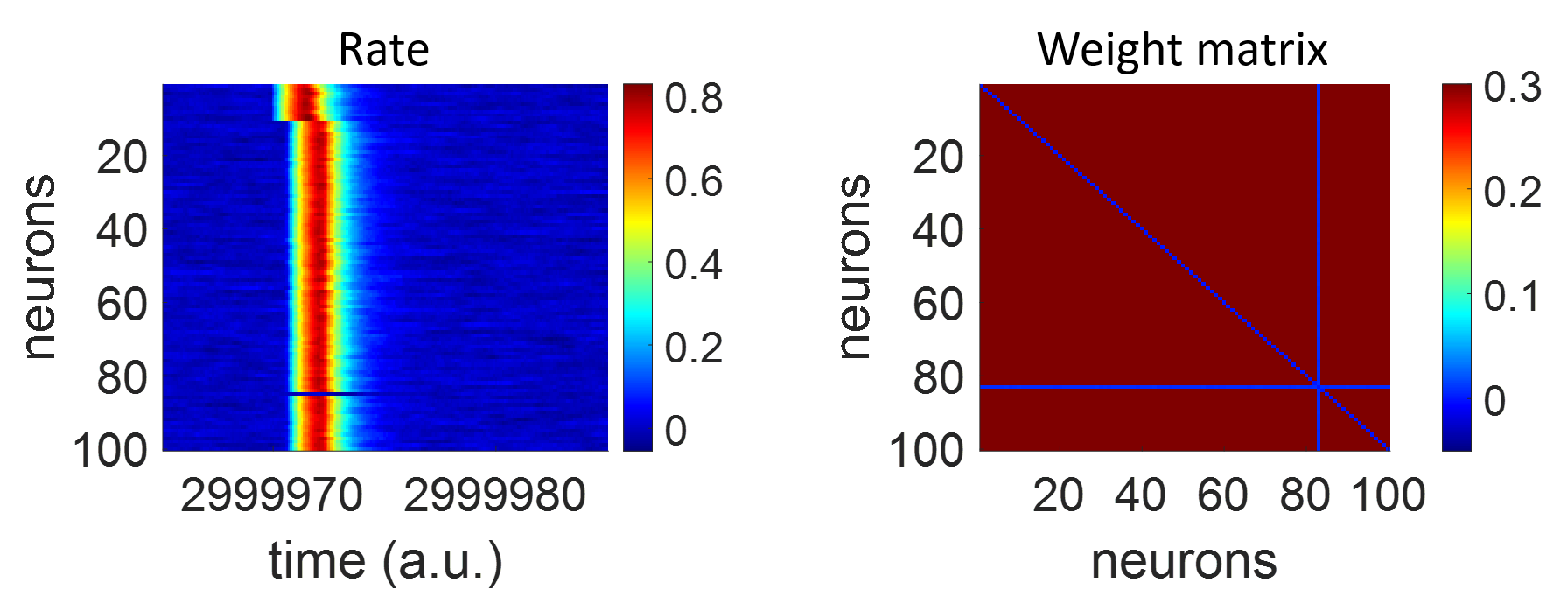

Supplement: S2 Fig — Starting at time 0 a.u., 10 neurons were stimulated for 100000 times with f = 1/(30 a.u.). The firing rate plot and the weight matrix show that almost all the network had been recruited into one assembly after 100000 stimulations. (TIF) [file pcbi.1011727.s003.tif]

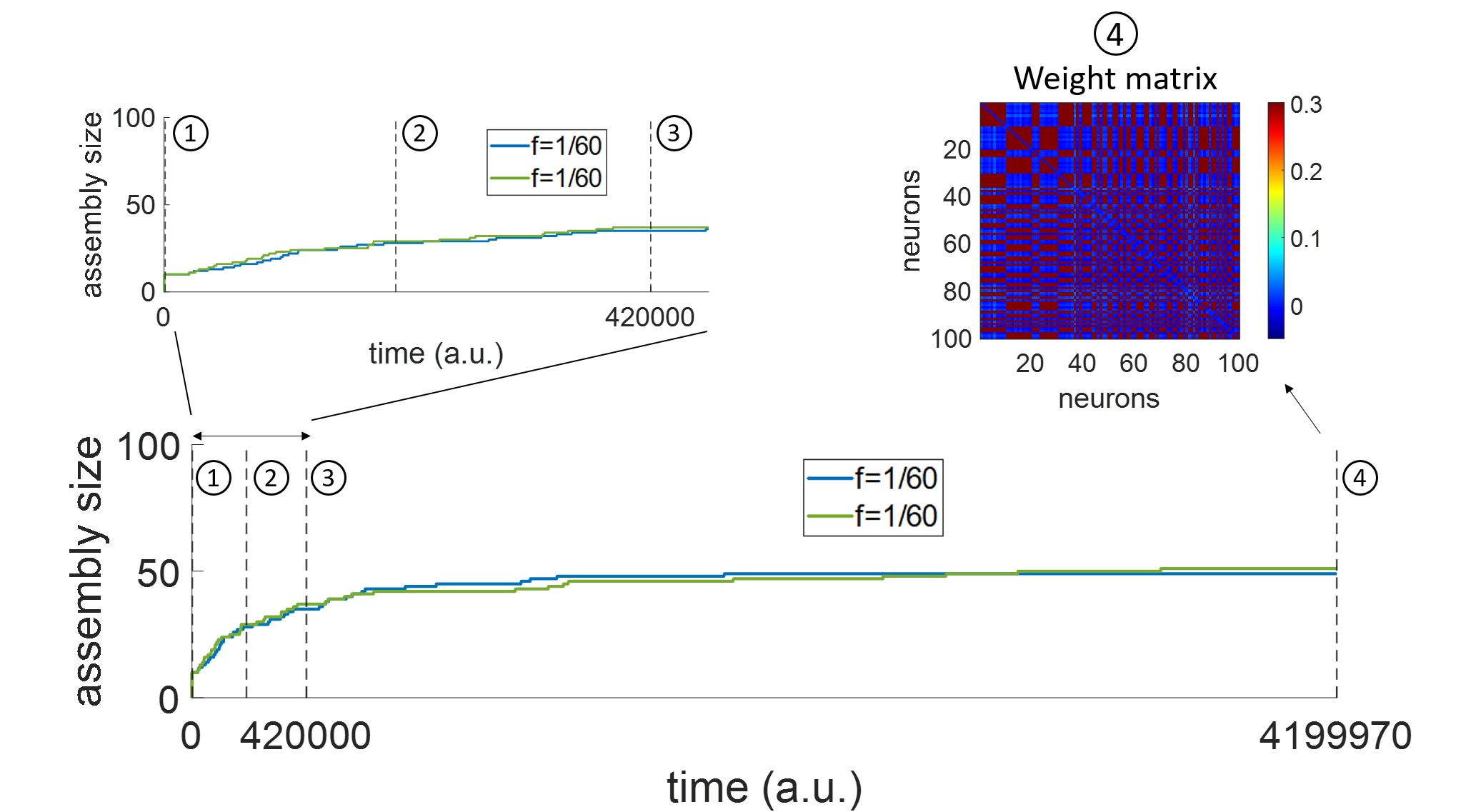

Supplement: S3 Fig — Starting at time 0 a.u., two non-overlapping populations of 10 neurons each were stimulated at different times with f = 1/(60 a.u.) for 70000 times. Bottom: number of neurons per assembly over time. Inset, top left: zoom with enlarged time scale. Top, right: weight matrix at the end of the stimulation paradigm. (TIF) [file pcbi.1011727.s004.tif]

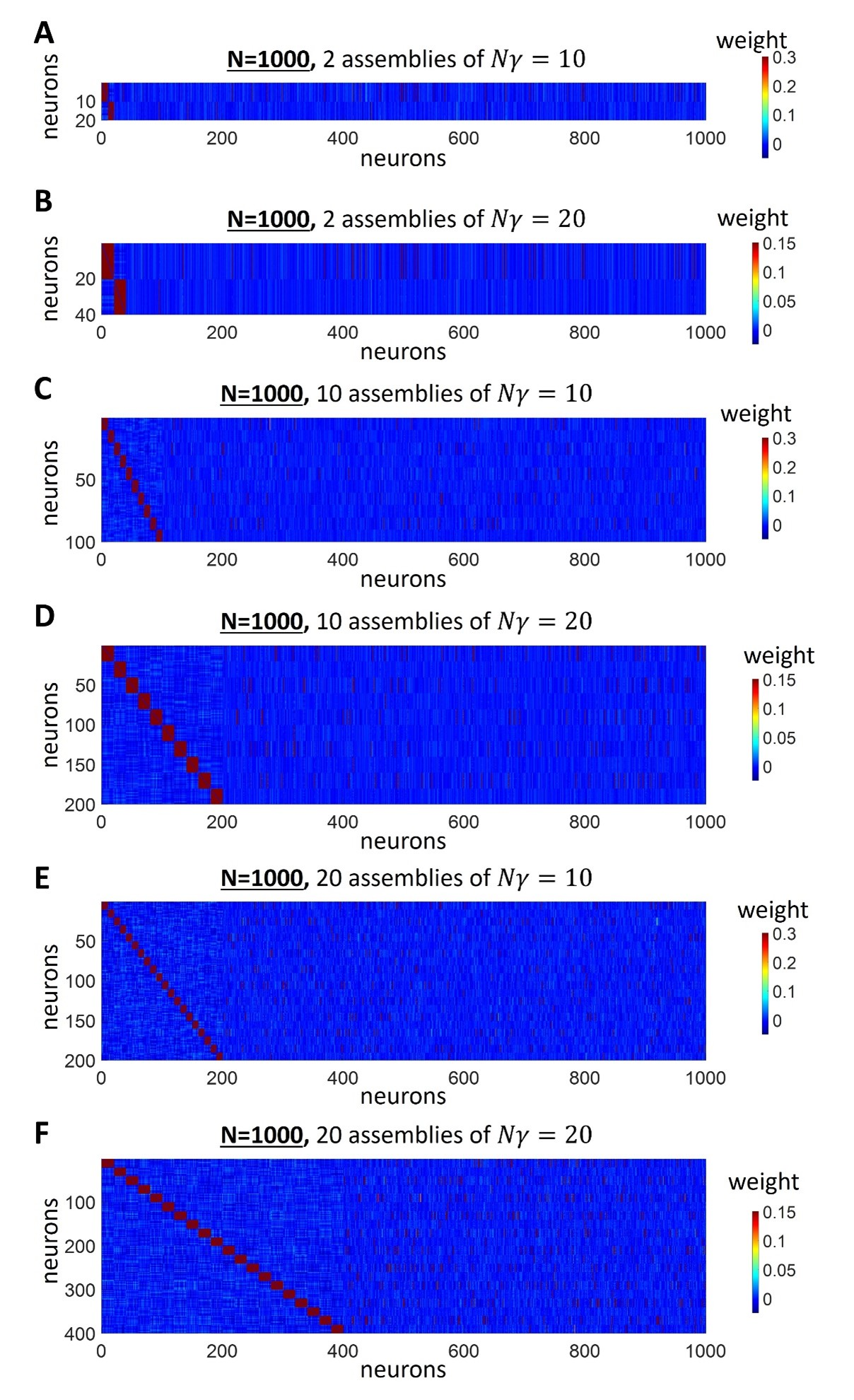

Supplement: S4 Fig — A-B-C-D-E-F) Connectivity, at t = 350000 a.u., among stimulated assemblies within networks of 1000 neurons, in case of different number of assemblies and number of stimulated neurons, namely: A) 2 assemblies of 10 stimulated neurons (N ∙ γ = 10); B) 2 assemblies of 20 stimulated neurons; C) 10 assemblies of 10 stimulated neurons; D) 10 assemblies of 20 stimulated neurons; E) 20 assemblies of 10 stimulated neurons; F) 20 assemblies of 20 stimulated neurons. In each simulation, two stimulation frequencies (f1 = 1/(600 a.u.) and f2 = 1/(1200 a.u.)) were used, with half of the assemblies stimulated with f1 and half of the assemblies stimulated with f2. For better visualization, only connections in one direction are shown (however, reciprocal connections have the same value). Connections among the non-stimulated neurons are not shown. In none of the simulations we observed the formation of overlaps between different assemblies. (Parameters: β = 0.000125.). (TIF) [file pcbi.1011727.s005.tif]

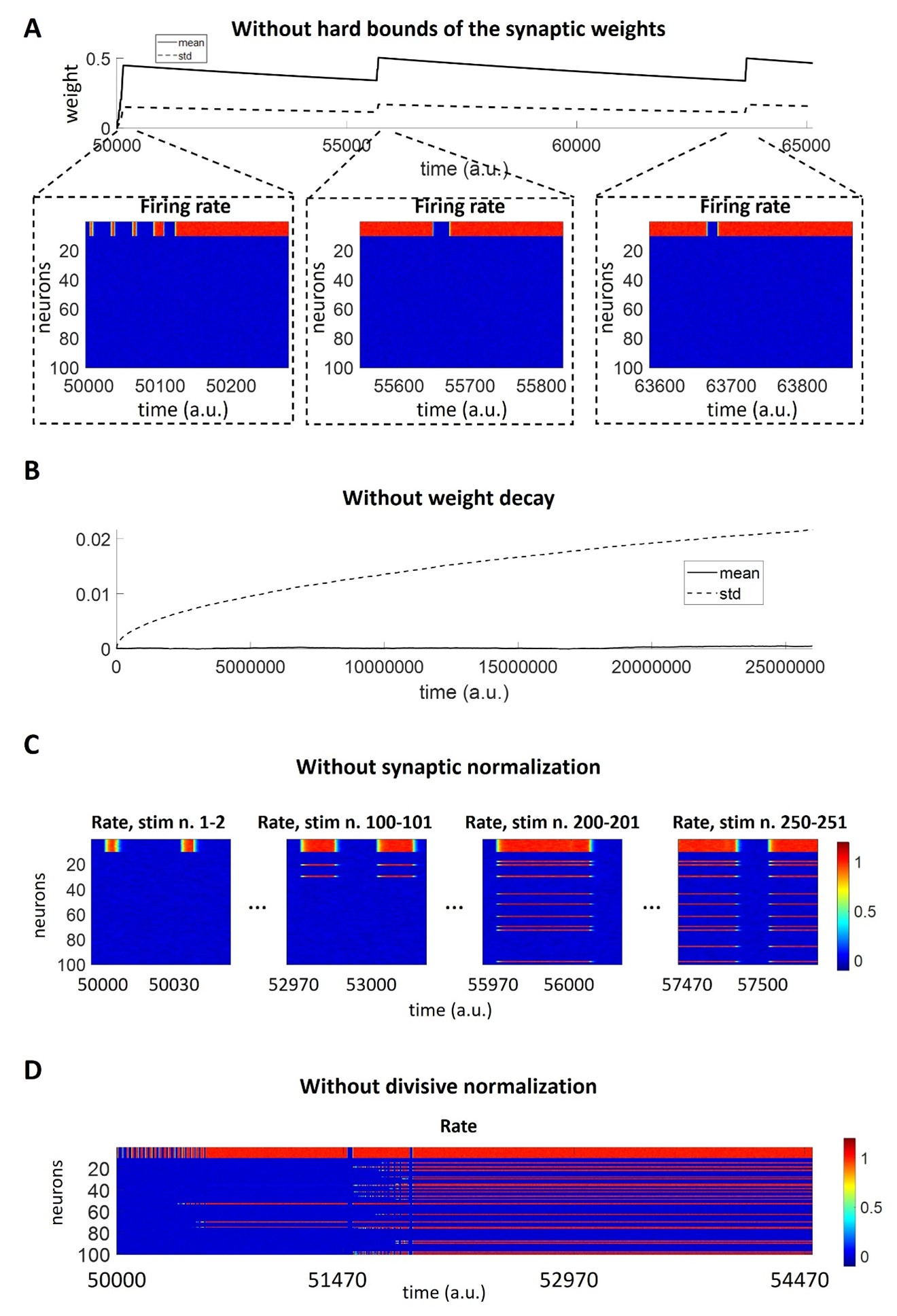

Supplement: S5 Fig — A) Model without hard bounds of the synaptic weights. A specific population of 10 neurons was stimulated repeatedly at a repetition frequency f=130a.u.. The mean weight of the network increased without limit until the stimulated neurons would not return to baseline activation after the end of the external stimulation. The weight successively decreased, due to forgetting and due to the fact that sustained co-activation for longer than TLR (i.e. length of the window adopted for calculating the learning rule’s running average; see Table 1 in the main text) does not result in learning (see Eq 8 in the main text). B) Model without weight decay. The mean and standard deviation of all network connections in absence of any external stimulation are displayed in the case of model without forgetting. Without forgetting, the standard deviation kept increasing, even if the other stability mechanisms were implemented. (Parameters: β = 0). C) Model without synaptic normalization: A specific population of 10 neurons was stimulated repeatedly at a repetition frequency f=130a.u.. The firing rates for all network neurons at different times are displayed. In absence of synaptic normalization, the formation of new connections with new recruited neurons lead to more prolonged assembly activation, eventually leading to uncontrolled network activity. (Parameters: αw = 0; αr = 2). D) Model without divisive normalization. A specific population of 10 neurons was stimulated repeatedly at a repetition frequency f=130a.u.. The firing rates for all network neurons are displayed, showing that, in absence of divisive normalization, the formation of new connections with new recruited neurons lead to uncontrolled network activity. (Parameters: αw = 2; αr = 0). (TIF) [file pcbi.1011727.s006.tif]

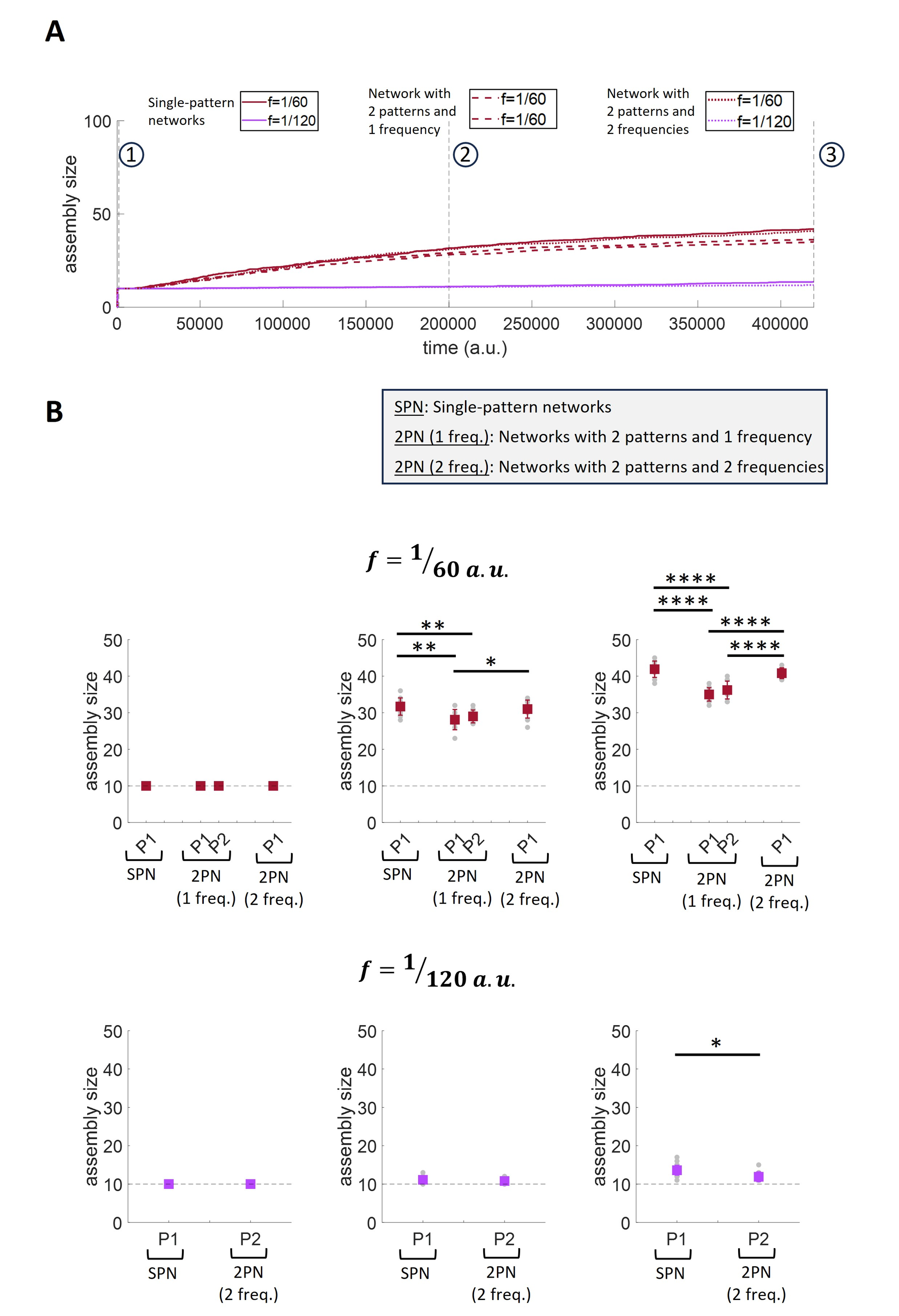

Supplement: S6 Fig — A) Number of neurons per assembly over time in case of “Single-pattern networks”, “Network with 2 patterns and 1 frequency”, “Networks with 2 patterns and 2 frequencies”. B) Number of neurons per assembly at different times for each of two stimulation frequencies (f=160a.u.; =1120a.u.) in three different experimental paradigms. It should be noted that the assemblies’ names “P1” and “P2” correspond, for each paradigm, to the ones used in the figures of the main text (Figs 6A, 7A and 8A). (TIF) [file pcbi.1011727.s007.tif]
