# Supplementary material for: A dynamic attractor network model of memory formation, reinforcement and forgetting
Source: PLoS Comput Biol. 2023 Dec 20;19(12):e1011727. doi: 10.1371/journal.pcbi.1011727 (PMC10766193; doi:10.1371/journal.pcbi.1011727)
Supplement: Attachment — Submitted filename: Reply_to_reviewers.pdf [file pcbi.1011727.s008.pdf]

Reviewer #1: Boscaglia et al. designed a Hebbian network with an online learning rule, random background activity, and biologically plausible dynamics to model the phenomena that familiar memories recruit more neurons while inactive memories are gradually forgotten. I found the paper well-written with an easy-to-follow exposition. The model design choices are minimal and reasonable, including the adaptive firing rate threshold, weight decay-based forgetting, and normalization rules. The results are clearly explained in terms of the model design. I have no essential concerns regarding the technical soundness of the model or the results.

We thank the reviewer for the positive feedback. We are happy to see that he/she has not essential concerns regarding our model and the results, and address all the comments below.

On the other hand, it is not entirely clear what conceptual insights or experimentally testable hypotheses the proposed model offers. The model is explicitly designed to capture dynamic aspects of memory beyond the remit of standard Hebbian networks and, as such, may be seen as a proof-of-concept of the authors' working hypothesis. Beyond this, the in silico results are descriptive and confirmatory in nature. Many results, such as forgetting, are direct consequences of the model design. Others, such as the enlargement of memory ensembles by recruiting randomly co-firing neurons or the orthogonality between different memories, are not entirely trivial yet still closely tied to specific design choices (noisy background activity and "synaptic normalization," respectively), as the authors also highlighted. It is not clear what experimentally testable hypothesis the model proposes, nor did the authors put forward any. Some model properties seem already incompatible with experimental results: not all memories are orthogonal, and the same hippocampal neuron may respond to multiple stimuli (the authors acknowledge this in the Discussion as needing further study). A model need not capture every experimental detail, but it should offer non-obvious insights or inform future experiments.

We agree with the reviewer that our work offers a modelling implementation to replicate experimental results (and could be seen as a proof of concept) and that many results are either direct or non-trivial consequences of the model design. The particular hypothesis that we tested - namely, that the size of a memory representation depends on the frequency of stimulation - is described in the introduction and it is illustrated in Figure 1. With respect to an experimental testable hypothesis, based on these results we predict that the repeated presentation of novel stimuli (e.g. an unknown person) should lead to the formation of gradually larger assemblies, which, due to the sparse sampling of single neuron recordings (and of the assemblies responding to the stimuli), should give an increasing tendency to find single neuron responses to novel stimuli with stimulus repetition. This is now mentioned in the 4th paragraph of the discussion.

With respect to the apparent incompatibility of the model with the experimental results, we have clarified this in the discussion, where we now say:
"Memory relies on the coding of associations - for example, to remember meeting a particular person in a particular place - a process that involves the hippocampus (Eichenbaum, 2004; Quian Quiroga, 2012; Wallenstein et al., 1998). Based on results obtained with single cell recordings in the human hippocampus, namely, that if a neuron responds to two or more concepts, these concepts tend to be associated (Rey et al., 2018; De Falco et al., 2016; Ison et

al., 2015), we suggested that in this area associations are encoded via partial overlaps between the assemblies representing the concepts involved (Quian Quiroga, 2012; Quian Quiroga, 2019). In a previous modelling study (Gastaldi et al., 2021), we have shown that such partial overlaps are efficient to encode and retrieve associations. This model assumes neglectable amounts of overlaps between non-associated items and it is therefore important that non-associated items (as the ones in this study) form non-overlapping representations, or otherwise, overlaps that may have been created by chance would be erroneously interpreted as encoding meaningful associations."

The authors' main working hypothesis is that familiar concepts have larger memory ensembles due to background firing activity that happens to co-occur with the stimulus. An alternative hypothesis, which seems to me conceptually distinct, is that a frequent concept is experienced under more contexts than an unfamiliar one; the diverse contexts may then be incorporated into a large memory assembly through Hebbian learning. This alternative is in fact equally well described by the authors' model if "background" activity is re-interpreted to mean not just random activity, but any activity concurrent with the remembered experience. It is not obvious whether the authors intend to accommodate this interpretation (and what alternative hypotheses the model would be incompatible with).

We agree with the reviewer that, besides the mechanism we have described, an alternative possibility is that familiar assemblies can be also enlarged by incorporating neurons representing contexts where the specific memory is experienced. In the corrected version we mention this possibility in the penultimate paragraph of the discussion, where we now say:

"We should also mention that an alternative (and not mutually exclusive) model for enlarging familiar assemblies is that, via partial overlaps, the assembly representing a concept that is experienced in different contexts may incorporates neurons representing these contexts, given that the context where a concept is experienced (i.e. a particular place) is another concept with a corresponding assembly."

Minor concerns

Equation 4: Is it possible for the firing rate threshold to decay asymptotically toward 0, or is it lower-bounded at theta_0? Related, when describing Fig. 4C, the authors refer to a threshold of 0.15. It is a bit confusing since 1) the threshold is dynamically changing and 2) none of the inputs in the histogram actually reach 0.15.

The threshold changes with the firing and tends to a constant value (Theta_0), which is different from zero. We believe that there is a typo and the reviewer refers to Figure 7C (not 4C) and note that in this figure we have previously excluded, for any unit later recruited into either P1 or P2, the trials after its recruitment, which are the ones with higher values of input field (if a neuron is recruited into an assembly, it will have relatively high input field values in the trials in which its assembly is stimulated). However, in this figure there are still values that reach 0.15 but they are relatively few and were therefore not visible in the previous version of the figure (we have now added an inset where we zoom on the higher input field's values). Then, we agree that it is confusing to refer to theta_0 since the firing threshold is dynamically changing and we removed the reference to a threshold of 0.15 in the description of the figure.

Page 5, last paragraph: Rather than "rather than," it seems more accurate to say "both...and".

We have slightly edited this sentence to avoid confusion. In particular, we now say: rather than **solely** to the number of stimulations.

Page 8, third paragraph: It is somewhat misleading to describe the assembly size increase as "larger" for the sparser network since the graph shows a relative size increase. Should not the absolute size increase be larger for the denser network?

We thank the reviewer for this comment and to avoid misunderstandings we now say that the increase is "relatively larger".

Page 9, third paragraph, fourth sentence: The sentence is very confusing. Do you mean that the orthogonal memories modeled here must only correspond to non-associated memory items?

Yes, that's exactly what we meant. We have expanded this sentence into a full paragraph to clarify this idea (paragraph before last in the discussion).

Fig. 7A is described at some length. However, is it not trivial due to the symmetry between the two patterns? The only difference is that pattern 2 always lags pattern 1 by t=30, which is expected to be a negligible difference.

In principle it is conceivable that there could be a 'winner takes all' dynamic, in which an assembly starts having a progressively larger increase and eventually recruits neurons from the other one. This is discussed in the fifth paragraph of the discussion and in the corrected version we have also added a short mention of this in the description of Figure 7A, where we now say:
"We were particularly interested in investigating if initially orthogonal neural assemblies would remain orthogonal when they evolve, or if, alternatively, one of the assemblies would show a progressively larger increase and eventually recruit neurons from the other one, showing a 'winner takes all' dynamics."

Fig. 7C, right: the x-axis labels are too closely spaced and thus a bit confusing

We have spaced more the labels to avoid confusions.

Reviewer #2: In this intriguing paper, the authors investigate the dynamics of memory encoding in the ecologically-relevant scenario in which different stimuli are encountered with different probabilities (per unit time).
In the standard attractor model, as commonly used to model memory storage and retrieval, the neurons encoding for the different memories (asymptotically) are chosen randomly with a fixed probability and the resulting memories are embedded with uniform strength into the synaptic matrix. This is, clearly, an oversimplified description, which, in fact, does not appear to be fully consistent with experimental observations in the medial temporal lobe (as discussed at length in the manuscript).

We agree with the reviewer that the standard attractor model is an oversimplified description of memory functioning and to this end we have introduced several modifications to this model, considering the dynamic nature of memory formation, reinforcement and forgetting.

To study how memory encoding depends on the history of the stimulation, the authors numerically investigate a model network that features several physiologically-grounded plasticity mechanisms. The dynamics of the model network, under different protocols of stimulation, are carefully dissected.
The authors report several interesting, and in my opinion important, results. In particular: (i) the number of neurons encoding for a particular stimulus increases as a function of the frequency with which the stimulus is encountered; (ii) neurons in an "expanding" neuronal representation are recruited among non-responsive neurons, so that initially non-overlapping representations will remain non-overlapping; (iii) neurons belonging to "forgotten" memories will be automatically made available again to participate in other memory representations.
The study addresses a key issue in memory function (i.e., how memories are dynamically updated) and presents novel and important results that significantly add to the credibility/plausibility of the attractor framework. The paper is clearly written and figures are very informative. I do not hesitate in recommending publication.

We thank the reviewer for the precise summary of our findings, the positive feedback, and for recommending publication.

Reviewer #3: The paper addresses the empirical observation that human memory representations are larger for familiar ones than unfamiliar items. The authors hypothesize that familiar representations undergo multiple repeated reactivations. This causes neurons firing with background rates, outside the original memory assembly, to form strong synaptic connections with the assembly, through ongoing Hebb plasticity rule. Several mechanisms are invoked to stabilize the system and prevent runaway of assembly sizes, connectivity strength and neuronal activity. The paper incorporates these hypotheses in a detailed recurrent network model and demonstrates through computer simulations the working of the hypothesized mechanism for the growth of memory assemblies.
The main hypothesis of the paper, as outlined above, is reasonable. However, the paper suffers from major limitations.

We thank the reviewer for the feedback. We are happy to see that he/she considers the main hypothesis of our paper reasonable and address all his/her comments below.

1. The conclusions are based on the outcome of computer experiments of the network model, with virtually no analysis. It is thus, very difficult to assess the generality, robustness and scalability of the proposed mechanism, and many questions remain.
For instance, it is unclear what governs the decay time of the different assemblies, beyond the displayed trajectories of the assemblies. Naturally, the results should be cast in the form of histograms of assembly sizes and life times derived from large scale simulations. Scalability is demonstrated by using two sizes (and the same number of assemblies). No attempt is made to scale the number of memories with network sizes.

The reviewer says that our paper shows virtually no analysis and that it is thus very difficult to assess the generality, robustness and scalability of our results. We respectfully disagree. In particular, we have demonstrated consistent results with different number of patterns, different stimulation frequencies, different number of units and assembly sizes, etc.

With respect to the decay time, it depends basically on the forgetting term, as shown in Figure 3 and regarding the presentation of the results, in most cases we show the evolution of the assemblies and the mean and SEM values at different times, thus characterizing the distributions.

Following the reviewer's suggestion, in Figure 10 we now describe results with N=500 and N=1000 (using in each case two different frequencies of stimulation and two assembly sizes), obtaining results consistent with those described in the rest of the paper and showing the scalability of our results. Furthermore, we have added a new figure, Figure 11, where we show results with N=1000 and different combinations of number of assemblies and assembly sizes (specifically, 2 assemblies of 10 neurons, 2 assemblies of 20 neurons, 10 assemblies of 10 neurons, 10 assemblies of 20 neurons, 20 assemblies of 10 neurons and 20 assemblies of 20 neurons) for two different frequencies. As described in the section "Scalability" in Results, we obtained results consistent with the rest of the work.

Finally, we should remark that it was not our intention to investigate the network capacity, by e.g. systematically varying the number of memories for different network sizes, which is

something that has been extensively described in the literature and it is beyond the scope of our paper.

2. Notably, most of the results are for very long time in arbitrary units or number of repetitions. No attempt is made to translate to biological or behavioral time scales. Are the number of repetitions reasonable?

We used relatively long simulation times to show convergence of the results (i.e. that assembly sizes don't keep increasing without limit). In general, it is very difficult to objectively define biological or behavioural time scales, as these depend on many factors such as emotional saliency, context, association with other memories, etc. In our model the timescales depend basically on the learning rate (constant η in equation 7), as well as on level of background activity and synaptic decay rate (constant β in equation 8). Note that with our parameter choices, the assemblies were consolidated within about 5 repetitions (Figure 4), which seems reasonable (i.e. not saturating at the first presentation, thus showing the effect of consolidation, but also not taking way too many trials). However, we remark again that it is difficult to define what is a reasonable speed of consolidation, and our model gives the flexibility to vary it with the abovementioned parameters.

3. The model adopts multiple stability mechanisms (hard bound on synaptic weights, neuronal divisive normalization, synaptic divisive normalization) and weight decay. Are all these necessary for stabilizing the system?

Yes, these implementations are indeed necessary to stabilize the system.
We have now added Figure S5 where we show what happens if removing each of these stability mechanisms (In Figure 2 of the previous version we had already showed the progressive introduction of the background activity, the weight decay and the normalization mechanisms). Furthermore, we have now added references related to the choice of hard bounds of synaptic weights in the methods section (Zenke et al., 2017; Gerstner et al., 2002; Rubin et al., 2001; Gütig et al., 2003).

4. The paper completely ignores the question of retrieval. Are these assemblies dynamic attractors? What would be the retrieval mechanisms and how it will be affected by the enormous imbalance between assembly sizes? What do the neurons in the assembly code? In most memory models, each assembly code for high resolution information embedded in the pattern, but as the assembly grows in size in a stochastic manner it is unclear what their activation during recall stands for?

Our model is still an attractor network model and the assemblies are indeed attractors, as shown extensively in our previous work (Gastaldi et al., PLOS Comp Bio 2021) and many other studies. Likewise, the retrieval mechanism is the same as for attractor networks – i.e. a pattern similar to the one stored in memory will lead to the retrieval of the memory patterns (this has been also discussed in detail in Gastaldi et al). This retrieval mechanism is also present with an imbalance between the assembly sizes, as in the cases shown in Figure 8, Figure 9 and Figure 11.

We link our model to findings with recordings of neuronal activity in the human medial temporal lobe, where it was found that neurons respond in an invariant manner to specific concepts (e.g. a particular person) and that familiar concepts correspond to larger assemblies compared to non-familiar ones. In this sense, neurons in the assemblies of our model correspond to specific concepts and provide a representation that changes with the frequency of stimulation (i.e. the familiarity of the stimuli), as found in the real data.

5. Relatedly, the assumed very extensive repeated stimulation of memory patterns as a reasonable model of familiar memories. It seems more plausible that at least in part, reactivation of memories is a result of an internal process which is self-supporting. This process has been studied in the literature (for instance, Lansner et al., Kreiman and Shaham) and seems to be ignored here. Thus, large assemblies can be the result of only a small imbalance in external rehearsal which then drives a self-sustained process of spontaneous reactivation.

We agree with the reviewer that consolidation can be also due to internal processes, such as the spontaneous reactivation of the assemblies, and thank the reviewer for the comment and the references. In the corrected version we have added a brief discussion about this in the 3rd paragraph of the discussion, including the references the reviewer mentioned as well as another related reference from Van Rossum's group.
