# Supplementary material for: A dynamic attractor network model of memory formation, reinforcement and forgetting
Source: PLoS Comput Biol. 2023 Dec 20;19(12):e1011727. doi: 10.1371/journal.pcbi.1011727 (PMC10766193; doi:10.1371/journal.pcbi.1011727)
Supplement: Attachment — Submitted filename: Reply to reviewers_2ndRevision.pdf [file pcbi.1011727.s009.pdf]

Reviewer #1:

As in my last review, I have no concerns with the soundness of the study. This evaluation does not change since the authors have made no new claims.
At the same time, my previous concern remains. I believe the study a) contributes a proof-of-concept model replicating experimental results but b) primarily describes the model (cf. biology). My reading of the authors' reply is that they acknowledge this characterization.
The journal serves both computational and biology audiences, so I think an ideal paper should use a computational model to derive a new biological insight or suggest a novel experiment. Therefore, I asked about a testable hypothesis for future experiments. I am not sure the authors fully addressed this request. The authors reply,
'[...] based on these results we predict that the repeated presentation of novel stimuli (e.g. an unknown person) should lead to the formation of gradually larger assemblies, which, due to the sparse sampling of single neuron recordings (and of the assemblies responding to the stimuli), should give an increasing tendency to find single neuron responses to novel stimuli with stimulus repetition.'
The reply seems to refer to the same hypothesis that motivated the model in the first place, not a novel prediction the model has uniquely allowed the authors to make. Indeed, the authors present the hypothesis as motivating the model, not vice versa.

We have edited this sentence as suggested by the reviewer.

This concern does not take away from the value of the study in offering a quantitative, working model of a simple hypothesis regarding a relevant question. Again, I appreciate the clean, minimal model and the paper's clear exposition. Meanwhile, I believe the revision has not increased the significance of the study to the discipline.
The authors can potentially improve the significance of the study by committing to specific modeling choices as biological hypotheses, thus turning the model properties resulting from those choices into testable predictions. The onus is on the authors to find predictions that are experimentally testable. For example, here are some non-trivial predictions one may derive from the model, but they are not easy to test:
1. Background activity is required to enlarge memory assemblies. This prediction seems difficult to test without a method to manipulate background activity.
2. Synaptic normalization (S_{w,i}) is required to keep memory assemblies orthogonal. This hypothesis also seems challenging to test. Is synaptic normalization biologically plausible? If so, is there an experimental way to manipulate (e.g., remove) synaptic normalization?

Following the reviewer's suggestion, we have proposed two testable predictions in the 4th paragraph of the Discussion:

"Our results show the role of synaptic normalization in reducing the firing of the neurons that are already recruited into an assembly, thus decreasing the probability to be recruited by other assemblies and keeping assemblies orthogonal. Based on this result, we can propose two experimental predictions: i) neurons with low firing rates are already part of consolidated memory assemblies and are therefore less likely to respond to novel stimuli (i.e. novel stimuli will tend to recruit neurons with relatively high firing rates); ii) the neurons that start firing to novel stimuli should decrease their baseline firing rates with the repeated presentation of the stimuli."

Reviewer 3 commented on the many stimulus repeats required to establish an assembly and the need to relate model time units to a realistic time scale. I understand the general difficulty of linking model parameters to physical units. I also appreciate that physical correspondences can be out-of-scope for a particular computational model (e.g., people do not expect deep neural networks to model membrane voltage dynamics). Nevertheless, if the authors could commit to an interpretation, that would add specificity to the model predictions and, thus, the paper's conceptual relevance.

We have added this information in the Results section (in the subsection "Assembly formation and reinforcement"), where we now say:

"Although it is very difficult to objectively define biological or behavioural time scales, as these depend on many factors (e.g. emotional saliency, context, association with other memories), it can be noted that the formation and reinforcement of the assembly within 5 repetitions of the stimulation seems reasonable - i.e. not saturating at the first presentation of the stimulus, thus showing the effect of reinforcing the assembly connections but also not taking way too many trials."

I believe the writing can benefit from more clearly delineating the properties of the model per se and the properties the authors expect to generalize to biology. For example, the abstract states,
'Specifically, we show that a dynamic interplay between Hebbian learning and background firing activity determines the relationship between the memory assembly sizes and their frequency of stimulation.'
I found this claim uncomfortable as it seems to draw a general conclusion, but the result is entirely about the model. In fact, the claim is partly tautological with the modeling assumptions ('Here we develop a modeling approach to provide a mechanistic hypothesis of how hippocampal neural assemblies evolve differently, depending on the frequency of presentation of the stimuli.'), so it adds little new beyond the proof-of-concept (i.e., the model 'works'). Try changing the word

'determines' to 'can explain'—the two ways of phrasing may illustrate the difference between committing to a biological hypothesis vs. designing a model to explain an observation.

To avoid misunderstandings we have edited this sentence as requested.

A similar comment applies to the remainder of the claims in the abstract: assemblies remain independent; unstimulated connections are forgotten. Each is a direct consequence of a modeling choice (synaptic normalization; weight decay). Because modeling choices are (in principle) alterable, what should we learn from the study beyond the properties of the specific model?

We agree with the reviewer that the claims in the abstract are direct consequences of our modelling choices (some more obvious than others, as discussed in the previous round of comments). However, we believe that it was not obvious that the modelling implementation we describe would lead to these results and support our main working hypothesis - i.e. that assemblies will increase or decrease their size depending on the rate of presentation of their corresponding stimuli. This is mainly because the use of the learning rule together with the background firing (compared to 'static' configuration of traditional attractor networks) imposed several challenges that required introducing normalizations, a forgetting term, etc, (as described in the Results section) to avoid that the dynamics of the network diverge by continuously increasing the mean firing of the network, recruiting all neurons into the assemblies, etc.

Minor suggestions
- The first two sentences in the abstract seem to make a logical leap. Why are larger representations necessarily easier to recall?

Following the reviewer's comment, we have slightly edited these sentences, which now read:

"Empirical evidence shows that memories that are frequently revisited are easy to recall, and that familiar items involve larger hippocampal representations than less familiar ones. In line with these observations, here we develop a modelling approach to [...]"

- Fig. 2A: The text first describes the two matrices on the right before those on the left. Could the authors arrange the figure and the text in the same order, to aid the reader?

We have arranged the figure and the text as suggested.

- Figs. 2A and 4C invite comparisons. Why does Fig. 4C (no connections beyond neurons 1–10 at t_10) differ from the right side of Fig. 2A (some connections beyond neurons 1—10 at t_15)? I guess it is because Fig. 4C had pauses between stimulations. However, Fig. 4A suggests to me that Fig. 4 used continuous stimulation. It would help to clarify how Figs. 2A and 4 differ.

The main difference between Fig. 2A and Fig. 4C is that in Fig. 2A there is no forgetting (i.e. $\beta=0$) and it is therefore easier for an assembly to recruit other neurons. We are not explicitly comparing these two figures because Figure 2 describes the details of the model implementation (with and without normalizations, forgetting term, etc.), whereas Figure 4 describes the assembly formation and reinforcement.

- Fig. 7A: Is this result expected based on symmetry? The authors reply that there could be a winner-take-all dynamic. What is the specific hypothesis—that P1 (the one slightly leading by t = 30 a.u.) will always win over P2? If the winner is stochastic from run to run, the t-test would still not be informative.

We agree with the reviewer and have removed the t-test. In Figure 7A we have also added the size difference between the 2 assemblies for each run, which doesn't increase and therefore reinforces the idea that there was not a winner take all dynamic.

- Figs. 7A, 8A: It would help to plot the conditions from Fig. 6A as a reference. I would expect that, e.g., two f=1/60 patterns competing for neurons in Fig. 7A would slow down assembly growth vs. Fig. 6A. It would be nice to be able to do this comparison.

We thank the reviewer for the suggestion and we have added this in Supplementary Figure (Fig. S6).

- 'Relatively larger': The authors made this change to address a previous minor suggestion, but I think it can be clearer. How about 'proportionally larger'? I am not sure about the interpretation that this difference is because 'as in this case there were more neurons that could be recruited into the pattern.' Again, the number of neurons recruited into the denser pattern is likely higher. The result could just be because the denominator is larger for the denser pattern.

We have changed "relatively larger" to "proportionally larger" as suggested. Note that in each curve of Figure 10 the denominator is constant and therefore the different slopes observed when neurons are recruited cannot be due to different denominators.

- The Results section does not mention heterosynaptic normalization, even though

it is featured prominently in the abstract. I suggest the authors introduce this model design when first describing the orthogonality result (Fig. 7).

Following the reviewer's suggestion, we now explicitly refer to the synaptic normalization when describing Fig.7.

We now say:

"The result of maintaining orthogonality between patterns is a consequence of the synaptic normalization - implemented in the model with the factor $S_{w,i}(t)$ - which normalizes the input field of each neuron according to the summed connection weight."

- There is a typo in 'may incorporates'

Thanks for pointing this out. We have corrected the typo.

Reviewer #2:

The quality and clarity of the manuscript, already very high in my opinion, have further improved with the present revision.

We thank again the reviewer for the positive feedback.
